# GATA1: A key biomarker for predicting the prognosis of patients with diffuse large B-cell lymphoma

**DOI:** 10.1515/med-2025-1185

**Published:** 2025-05-07

**Authors:** Yuxin Zhang, Yue Wang, Shifen Wang, Dawei Cui, Zheng Wei

**Affiliations:** Department of Hematology, Xiamen Branch, Zhongshan Hospital, Fudan University, Xiamen, China; Department of Hematology, Zhongshan Hospital, Fudan University, Shanghai, China; Xiamen Clinical Research Center for Cancer Therapy, Xiamen Branch, Zhongshan Hospital, Fudan University, Xiamen, China; The First Affiliated Hospital, Zhejiang University School of Medicine, Hangzhou, China

**Keywords:** bioinformatics, diffuse large B-cell lymphoma, GATA1, iron metabolism, ferroptosis

## Abstract

**Objectives:**

Diffuse large B-cell lymphoma (DLBCL) is a common and highly aggressive type of lymphoma. Iron metabolism plays a critical role in human diseases, however, which remains unclear in DLBCL patients. The study is to explore the genetic characteristics and molecular mechanisms underlying ferroptosis in DLBCL patients.

**Methods:**

Based on the GEO, GeneCards database, weighted gene co-expression network analysis was performed on the DLBCL sample and iron metabolism-related datasets. Enrichment analysis (Gene Ontology/Kyoto Encyclopedia of Genes and Genomes and gene set enrichment analysis) was used to analyze the expression and possible mechanism of key genes in DLBCL patients. The key genes were identified by quantitative real-time PCR.

**Results:**

The results showed that GATA-binding factor 1 (GATA1), as a key gene of iron metabolism in DLBCL patients, was related to the myeloid cell differentiation and granulocyte differentiation pathways to affect CD4^+^ T cells, B cells, and monocytes. GATA1 was strongly positively or negatively correlated with sensitivity to multiple targeted drugs. The patients with high GATA1 expression had shorter overall survival and worse prognosis than the patients with low expression. Additionally, high expression of the GATA1 gene was confirmed in DLBCL patients.

**Conclusions:**

GATA1 as one of the important genes of ferroptosis suggested a significant biomarker for predicting the prognosis of DLBCL patients.

## 1 Introduction

Diffuse large B-cell lymphoma (DLBCL) is a common and highly aggressive type of lymphoma, accounting for approximately 1/3 of all non-Hodgkin’s lymphoma cases [1]. In recent years, the incidence of DLBCL in China has been gradually increasing [2]. Because of the substantial heterogeneity of DLBCL, 30–40% of patients treated with the first-line immunochemotherapy regimen R-CHOP are either insensitive or develop relapsed/refractory lymphoma [3].

Iron is essential for maintaining normal function and homeostasis in cells. Therefore, an imbalance in iron metabolism is closely related to the occurrence, development, metastasis, and recurrence of many malignant tumors, such as lung cancer, renal cancer, breast cancer, prostate cancer, and liver cancer [4,5,6,7,8,9,10]. However, iron metabolism plays a dual role in tumor cells. On the one hand, the proliferation of tumor cells is more dependent on iron than that of normal cells, a phenomenon known as iron addiction [11]. Conversely, an increase in iron concentration leads to cell death through the accumulation of reactive oxygen species and lipid peroxidation products, known as ferroptosis [12,13]. Ferroptosis is a specific mode of programmed cell death that is dependent on iron metabolism and distinct from apoptosis, necroptosis, and autophagy [14]. With research on emerging anticancer pathways, various ferroptosis inducers have been developed for cancer [15,16].

Following the rapid development of gene microarray technology, researchers can measure the expression levels of thousands of genes in a short period, which helps gain a deeper understanding of the pathogenesis of diseases at the gene level. Currently, the International Prognostic Index is the main tool used for the clinical evaluation of DLBCL prognosis. Although such evaluations can guide clinical treatment, they merely provide a combination of clinical prognostic parameters that do not represent the heterogeneity of molecular biology in the occurrence and development of the disease. Therefore, it is critical to actively identify molecular biological indicators that affect DLBCL [17]. However, no previous studies have identified the genetic characteristics and molecular mechanisms underlying iron metabolism in patients with DLBCL.

To address this gap in the literature, we conduct bioinformatic analysis to identify the action pathway, core shared genes, and core genes of iron metabolism in the pathogenesis of DLBCL, thereby revealing the mechanism of iron metabolism and therapeutic targets in DLBCL. The results showed that GATA-binding factor 1 (GATA1) is a key gene in the role of iron metabolism in the pathogenesis of DLBCL. It affects the differentiation and maturation of immune cells through the myeloid cell differentiation pathway, especially memory B cells and CD4^+^ T cells, thus affecting the progression of DLBCL. The survival analysis found that the GATA1 high expression group had worse OS. Its expression had a positive correlation effect with BTK inhibitor-related drugs and a negative correlation with PI3K inhibitor-related small molecule drugs, which is meaningful for clinical drug selection. This research contributes to improving the early diagnosis, treatment, drug resistance, and prognosis of DLBCL.

## 2 Materials and methods

### 2.1 Data download and processing

We screened transcriptome sequencing datasets related to DLBCL using the Gene Expression Omnibus database. Iron metabolism-related datasets were downloaded from the GeneCards database (https://www.genecards.com/). Mutation information, clinical information, and genome-wide transcript levels of patients with DLBCL were obtained from The Cancer Genome Atlas (TCGA) database (https://portal.gdc.cancer.gov/). In addition, we obtained a list of all genes associated with immune response from the InnateDB database (https://www.innatedb.com/). Throughout the study, we background-corrected and normalized all raw data and matched all probe names with their corresponding gene symbols for subsequent analyses.

### 2.2 Weighted gene co-expression network analysis

Following scientific and technological advances and the rapid development of systems biology and bioinformatics, weighted gene co-expression network analysis (WGCNA) has become one of the most popular algorithms for analyzing large amounts of data. WGCNA addresses the correlation between gene sets and sample phenotypes, allowing for the mapping of inter-gene regulatory networks in gene collections and screening of essential regulatory genes closely related to disease [18]. In this study, we used WGCNA to establish a gene co-expression network for DLBCL and iron metabolism.

### 2.3 Identification and features of shared genes in DLBCL and iron metabolism

We counted the intersection of genes in clinically relevant modules to calculate the shared genes. TCGA data were used to verify the relationship between these shared genes and DLBCL and to observe their location on chromosomes and the frequency of copy number variations. GO enrichment analysis of shared genes was performed using the “ClueGO” and “MCODE” plug-ins in Cytoscape to establish protein–protein interaction (PPI) networks, understand their functions, identify the relationships between proteins, and ultimately screen out the core genes with the most critical roles. In this study, PPI construction was performed primarily using a STRING database (https://string-db.org/), whereby all available and predicted connections between proteins were integrated.

### 2.4 Functional enrichment analysis and gene set enrichment analysis (GSEA)

Gene Ontology (GO)/Kyoto Encyclopedia of Genes and Genomes (KEGG) enrichment analysis, which is the most common functional enrichment method currently employed in medical research, was used to interpret molecular-level information regarding the higher functions and functioning of biological systems. We applied the “clusterProfiler” R package to perform GO/KEGG enrichment analysis of shared genes and elucidate the underlying mechanisms of disease onset and progression. GSEA was used to analyze the distribution trend of genes in the predefined gene sets included in the gene list ranked by phenotypic correlation to determine their contribution to the phenotype. The median expression level of GATA1 was employed as a classification criterion to first categorize patients with DLBCL into low- and high-expression groups, before performing GSEA on the subgroups using GSEA software.

### 2.5 Association between core genes and DLBCL

We compiled gene expression profiles of DLBCL and normal tissues from TCGA and GTEx databases to compare the differential expression of core genes in DLBCL. The relationship between core genes and patient prognosis was determined by analyzing the clinical data of DLBCL in TCGA and calculating the overall survival in high and low GATA1 expression groups.

### 2.6 Assessment of the immune landscape

Tumor infiltration by immune cells can profoundly influence tumor progression and the success of anticancer therapies by exerting pro-tumorigenic and anti-tumorigenic effects. As a first step, we used immune cell infiltration and gene expression data from the TIMER database to calculate the relationship between core gene expression and immune cell abundance in DLBCL and presented these results in heatmaps and bubble plots. Second, we used single-sample genomic enrichment analysis, an emerging gene enrichment method, to compare the EstimateScore, ImmuneScore, and StromalScore between high- and low-expression groups of the core genes in each sample. In this calculation, we used gene set variation analysis to transform the expression matrix of individual genes into that of a specific set of genes. Finally, Spearman’s correlation analysis was used to analyze the relationship between core genes and a range of immune-related genes, such as immune checkpoint-related and immune cell subpopulation-related genes.

### 2.7 Quantitative real-time (qPCR) analysis

From February to April 2024, a total of nine adult inpatients with DLBCL were included in our study. The DLBCL diagnoses were confirmed through a tissue biopsy. As control subjects, nine healthy participants undergoing routine health examinations were included as health controls (HC). For each subject, 2 mL fresh blood was taken, and peripheral blood mononuclear cells (PBMCs) were isolated by FicollPaque density gradient centrifugation. Total RNA was extracted from each sample with TransZol Up Plus RNA Kit (TransGen Biotech, Beijing) and reverse transcribed (TransScript All-in-One Kit, TransGen Biotech, Beijing). The PCR analysis was done on an ABI StepOnePlus™ system using TransStart Top Green qPCR SuperMix (TransGen Biotech, Beijing) in triplicate. The 2^–△△Ct^ method was used to determine the relative expressions between HC and DLBCL, with GAPDH as the housekeeping gene. The primer sequences used in this study are listed in Table 1.

**Table 1:** Primer sequences of human GATA1 used in qPCR assay

Primers	Sequence (5′ to 3′)
Human GATA1 forward	CACGACACTGTGGCGGAGAAAT
Human GATA1 reverse	TTCCAGATGCCTTGCGGTTTCG

### 2.8 Statistical analysis

All analyses and visualizations were performed using R software (version 4.0.5). Kaplan–Meier survival curves were compared using the log-rank test. Unless otherwise stated, all *t*-tests in this study used *P* < 0.05 and logFold change|>2 as the criteria for statistical significance.


**Informed consent:** All participants gave informed consent before the start of the study.
**Ethical approval:** The clinical data of patients involved in this study were derived from an open-access database; therefore, consent was not required. All information regarding the ethical approval of these open-access databases can be obtained from two published studies. This study was approved by the Ethics Committee of the Xiamen Branch, Zhongshan Hospital, Fudan University (2023-XBZS-0236), and all study protocols complied with the Declaration of Helsinki.

## 3 Results

### 3.1 Selection of datasets

We selected two datasets of DLBCL from the Gene Expression Omnibus database, GSE83632 and GSE32918. GSE83632 was used for clinical trait analysis. This dataset contained a total of 163 whole blood samples; 76 specimens were blood samples from patients with DLBCL, which formed the experimental group, and 87 were from healthy individuals, which formed the control group. GSE32918 data were used to validate the results. Moreover, GSE32918 is derived from lymphoma tissues, so it can verify the expression of key genes in cancers. Six datasets related to iron metabolism were screened from FerrDB.

### 3.2 Screening of co-expression modules

**Figure 1:**
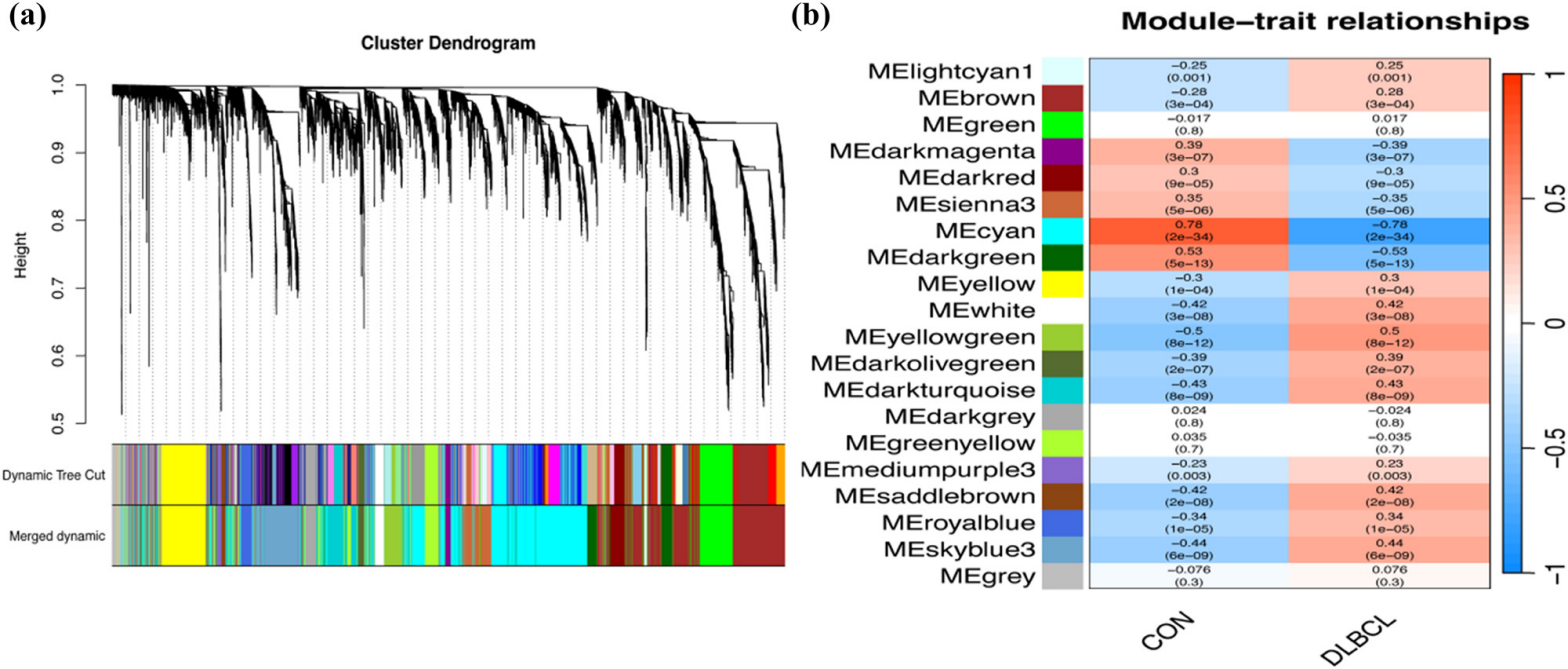
Identification of modules linked to clinical features of DLBCL. (a) Cluster dendrogram of co-expressed genes in DLBCL. (b) Heatmap of module–trait relationships in DLBCL.

WGCNA was used to analyze the GSE83632 dataset, with normal blood samples as the controls. We identified 16 gene modules that were closely associated with DLBCL, each of which is marked with a different color in Figure 1. Eleven gene modules were positively associated with DLBCL, of which the “dark green” and “ME cyan” modules were the most closely associated (dark green module: *r* = 0.5, *P* = 1 × 10^−11^; ME Cyan module: *r* = 0.78, *P* = 2 × 10^−35^; Figure 1(a) and (b)). These two modules were chosen as targets for subsequent analyses.

### 3.3 Screening of shared genes

**Figure 2:**
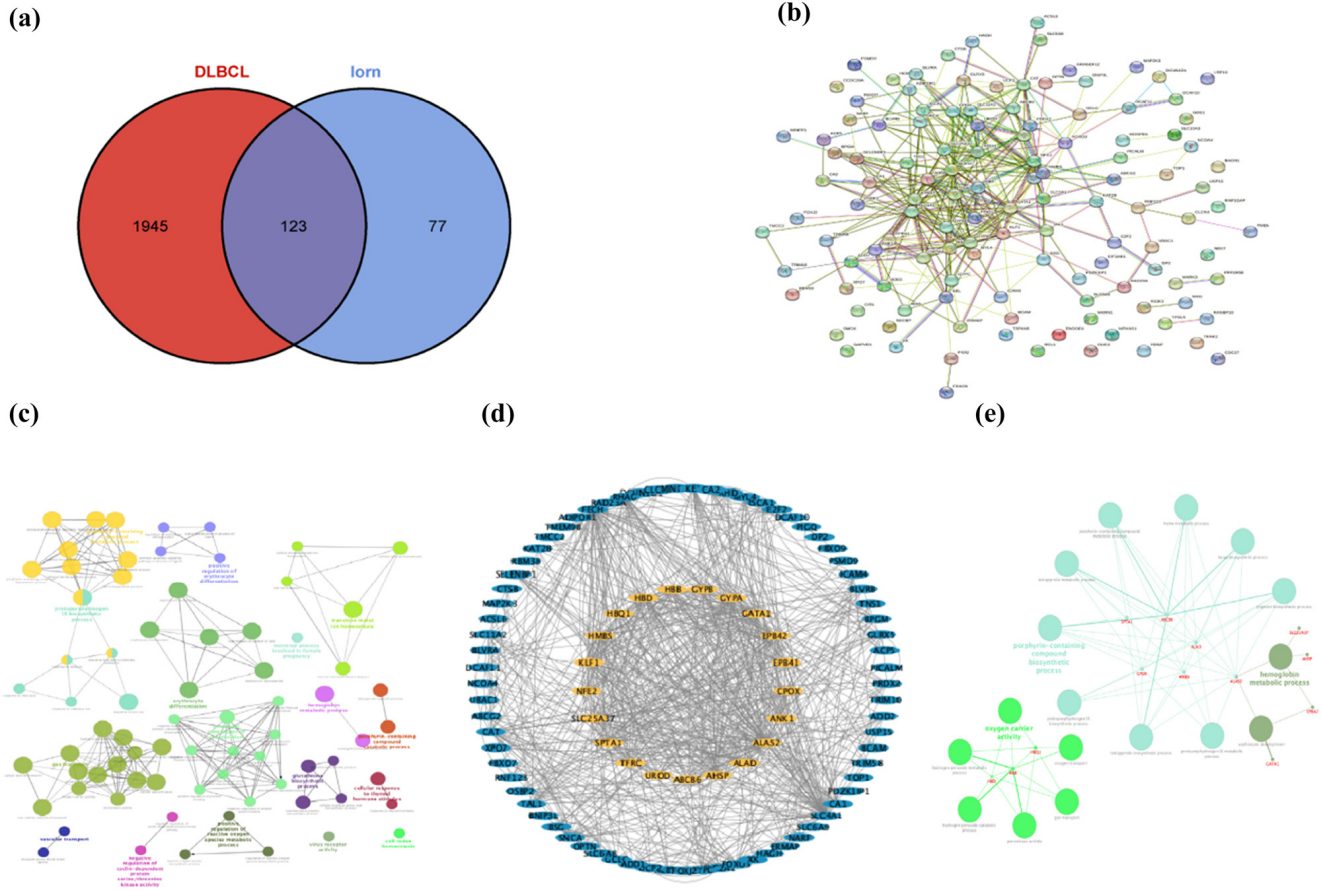
Characterization of shared genes between DLBCL and iron metabolism. (a) Venn diagram of shared genes between the two DLBCL modules and one iron metabolism. (b) PPI network of 123 shared genes. (c) Network of GO terms in ClueGO. (d) Network of MCODE. (e) Network of screened gene GO terms in ClueGO.

A total of 2,068 genes contained in the target module screened in the previous step were intersected with 200 genes related to iron metabolism to obtain 123 shared genes (Figure 2(a)). Using the interactions between these 123 genes, we constructed a PPI network (Figure 2(b)) to understand their interactions (Figure 2(c)). The network was imported into Cytoscape and the “MCODE” plug-in was used to filter genes with more than 20 chains, resulting in 21 shared genes (Figure 2(d)). The results of the “ClueGo” analysis were used to identify the potential mechanisms of these 21 shared genes in DLBCL, which are rich in biological activities, such as the apoptosis signaling pathway, oxidative stress pathway, and heme catabolic pathway (Figure 2(e)).

### 3.4 Validation of GATA1 core genes

**Figure 3:**
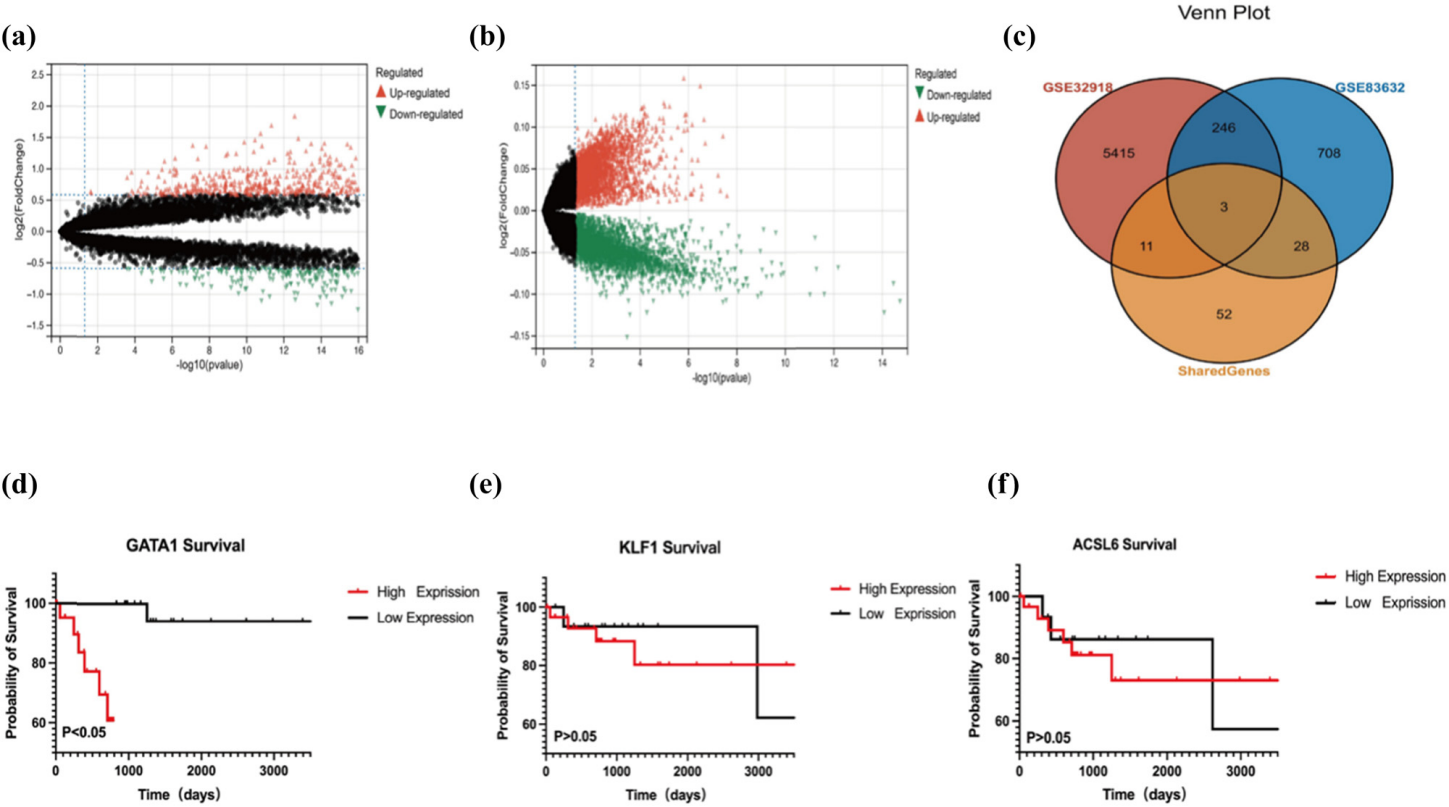
GATA1 expression and prognostic value in DLBCL patients. (a) Volcano plot of GSE32916 (log2 fold change|>1 and *P* < 0.05) and (b) GSE83632 (log2 fold change >1 and *P* < 0.05). Significantly upregulated and downregulated genes are depicted as red and blue dots, respectively. (c) Venn diagram of hub genes from the two DLBCL datasets and shared genes. (d) Kaplan–Meier curve of the association between GATA1 and overall survival in patients with DLBCL in TCGA datasets of DLBCL (*P* < 0.05). (e) Kaplan–Meier curve of the association between KLF1 and overall survival in patients with DLBCL in TCGA datasets of DLBCL (*P* > 0.05). (f) Kaplan–Meier curve of the association between ACSL6 and overall survival in patients with DLBCL in TCGA datasets of DLBCL (*P* > 0.05).

To ensure that the shared genes were expressed in both peripheral blood and tumor tissues and the accuracy of the screened shared genes, we applied differential analysis to DLBCL samples from the GSE32918 dataset and normal tissue samples, in which 5,675 highly expressed differential genes were screened from 24,527 genes (Figure 3(a)). Similarly, we applied differential analysis to analyze the differences between genes in DLBCL samples and normal tissue samples from the GS83632 dataset, in which 986 differential genes were screened from 11,023 genes (Figure 3(b)). By cross-analyzing the shared genes with the two differential genes, we obtained three shared genes with high reliability: GATA1, KLF1, and ACSL6 (Figure 3(c)). Considering DLBCL gene expression and prognosis, we identified GATA1 as the core shared gene involved in ferroptosis in DLBCL. In addition, the relationship between GATA1 expression and survival was analyzed using lymphoma-related data from TCGA. Patients with DLBCL and high GATA1 expression exhibited longer overall survival than those with low GATA1 expression (Figure 3(d)). Similarly, we analyzed the relationship between the expression levels of KLF1 and ACSL6 and survival in patients with DLBCL. There was no significance between them (Figure 3(e) and (f)). Survival and prognostic analyses were performed, and considering the gene expression and prognosis of DLBCL, we identified GATA1 as a core shared gene involved in ferroptosis in DLBCL.

### 3.5 GATA1-related biological processes and their associated signaling pathways

**Figure 4:**
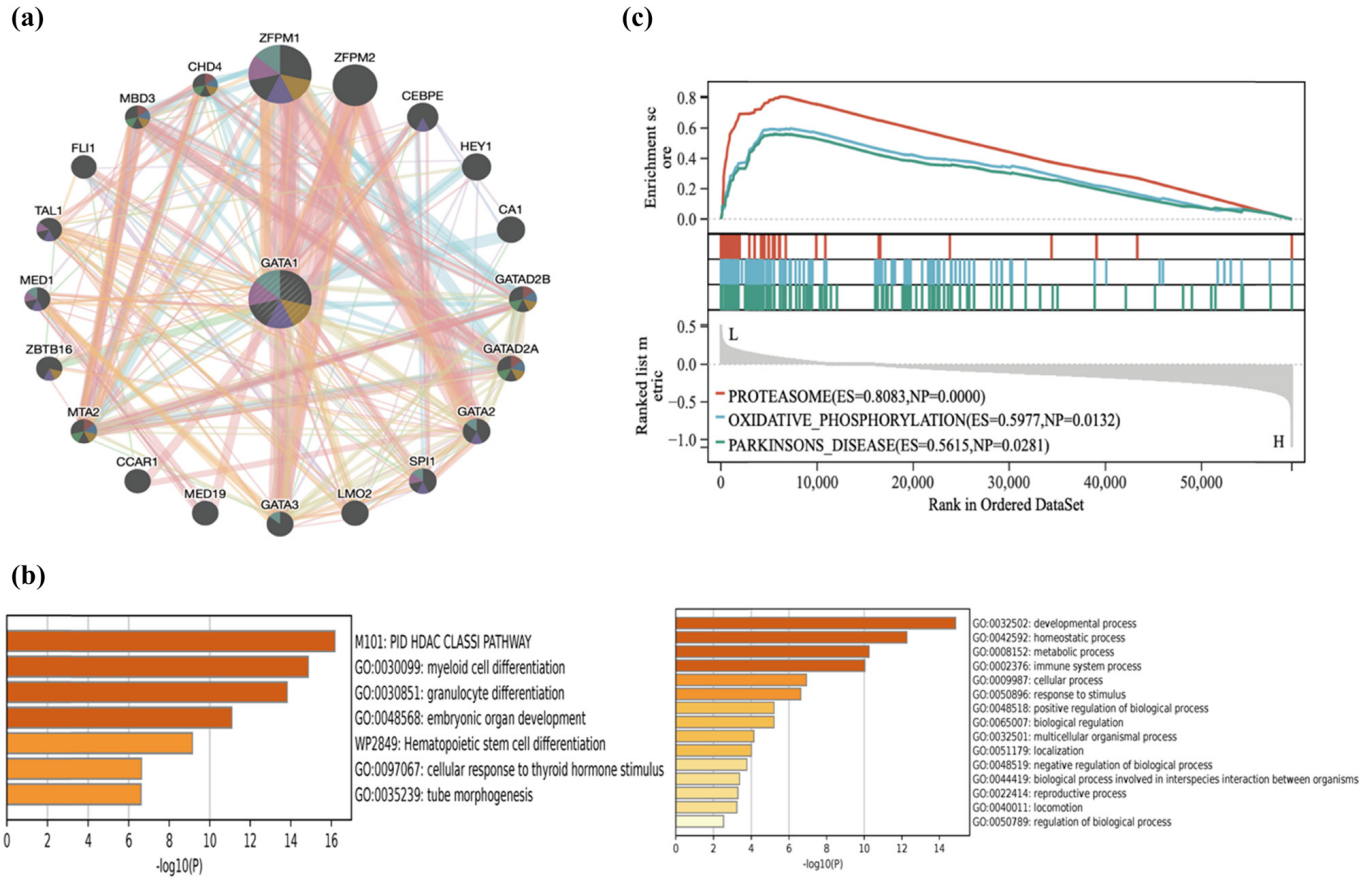
PPI network and functional enrichment analysis of GATA1. (a) PPI network of GATA1 and its interacting proteins. (b) GO/KEGG enrichment analysis of GATA1 and its interacting proteins (*P* < 0.05). (c) GSEA of the top 3 enriched pathways in patients with DLBCL and high GATA1 expression (*P* < 0.05).

After identifying GATA1 as a core gene, we explored the underlying mechanisms of GATA1 function using the GeneMANIA database (http://genemania.org) (Figure 4(a)), including analyzing the relationship between GATA1 and its genome-associated proteins, physical relationships, co-expression networks, and pathways, which provided a basis for our subsequent search for therapeutic targets. We performed GO/KEGG functional enrichment analysis of these genes, which turned out to be involved in the myeloid differentiation pathway, granulocyte differentiation pathway, embryonic organ development, and hematopoietic stem cell differentiation (Figure 4(b)). Because of the important regulatory role for cell differentiation, it occurred to us whether GATA1 could regulate the proliferation of DLBCL or could regulate the proliferation and differentiation of tumor-infiltrating immune cells, which could affect the therapeutic effect of DLBCL. Subsequently, we did GSEA of single gene GATA1 and found three pathways: PROTEASOME (*P* = 0.00 FDR = 0.0029), OXIDATIVE PHORYLATION (*P* = 0.013 FDR = 0.06), and PARKINSONS DISEASE (*P* = 0.028 FDR = 0.07), which can regulate the proliferation of DLBCL (Figure 4(c)). Combined with the current clinical situation, it is not difficult to find that many anti-DLBCL therapeutic drugs are realized through protease inhibitors, inhibition of oxidative phosphorylation, and GATA1 is closely related to these two pathways, and we hypothesize that GATA1 can be the next therapeutic DLBCL target.

### 3.6 Correlation between GATA1 and the immune microenvironment

**Figure 5:**
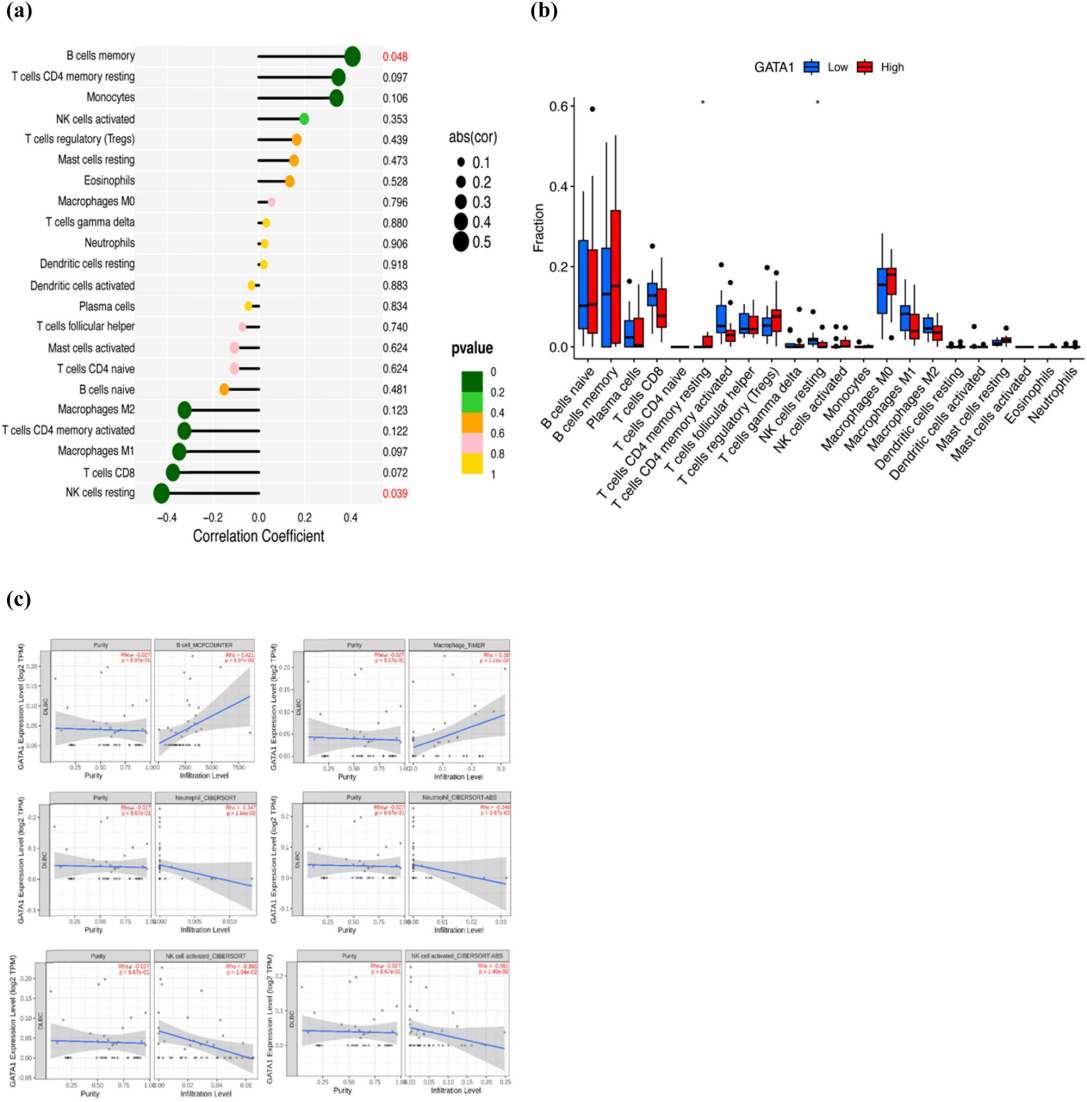
Distribution of immune cell infiltration in DLBCL. (a) Relationship between GATA1 expression and immune cell subtypes in patients with DLBCL (*P* < 0.05). (b) Immune cells between high-GATA1 and low-GATA1 groups. ssGSEA, single-sample GSEA. ns, no significance, * *P* < 0.05. (c) TIMER, CIBERSORT, and CIBERSORT ab algorithms were used to verify the relationship between GATA1 expression and B cells, monocytes, NK cells, and neutrophils.

**Figure 6:**
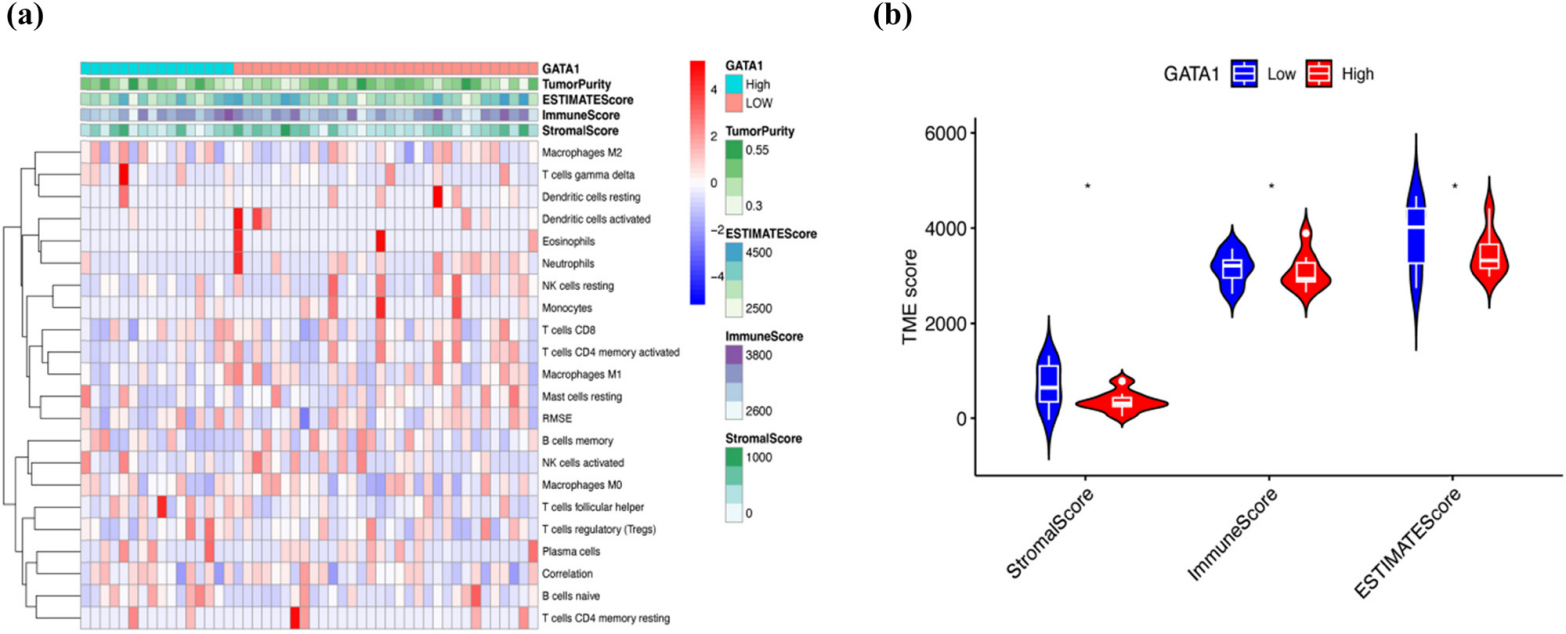
Immune microenvironment analysis in DLBCL patients with high/low LGALS2 expression. (a) Heatmap of immune cells between high- and low-expression groups. (b) Comparison of EstimateScore, ImmuneScore, and StromalScore between high-GATA1 and low-GATA1 groups using the ssGSEA algorithm. ns, no significance, *P < 0.05.

**Figure 7:**
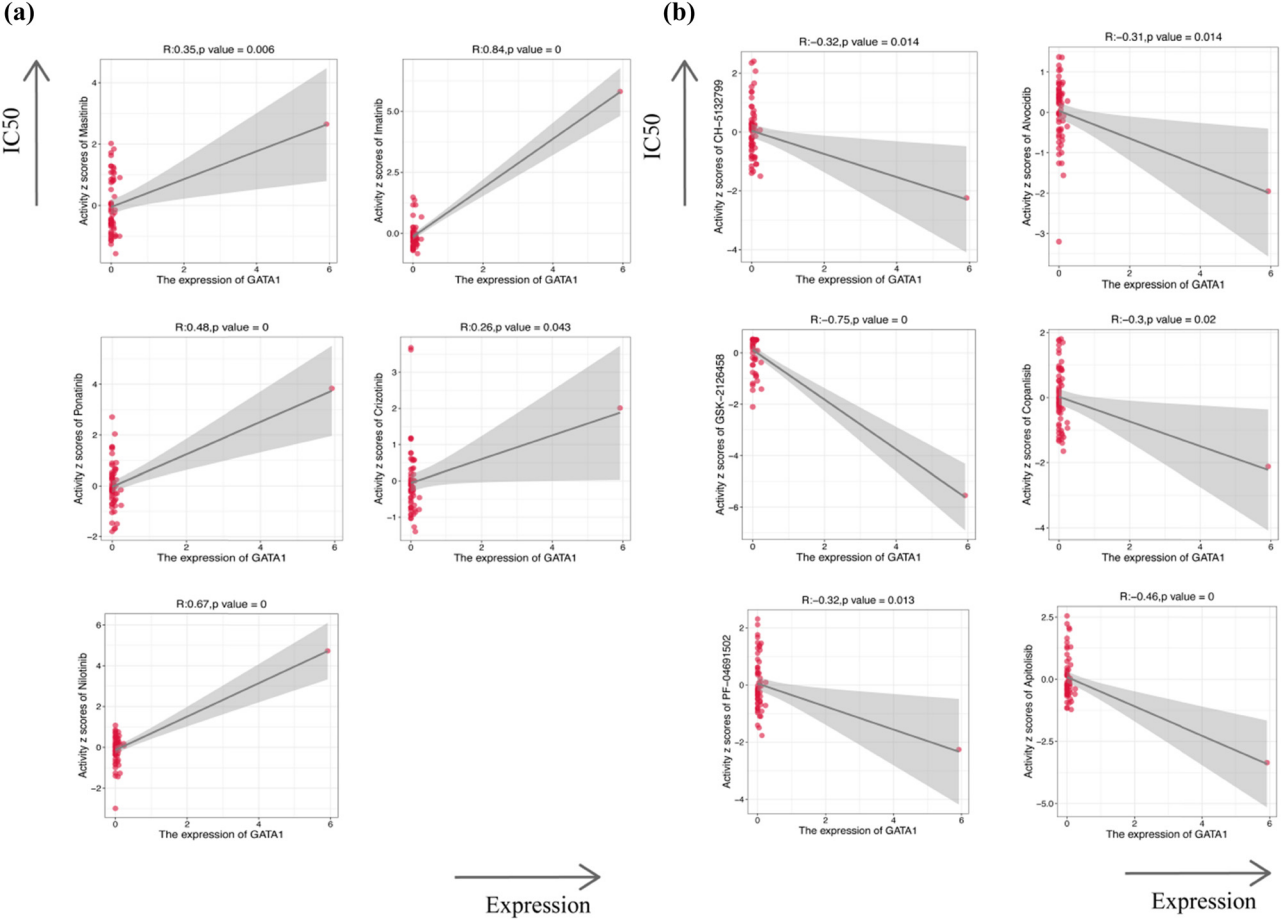
Drug sensitivity analysis of GATA1. (a) Drug sensitivity was positively correlated with GATA1 expression. (b) Drug sensitivity was negatively correlated with GATA1 expression.

We employed the single-sample GSEA (ssGSEA) algorithm to determine the relationship between GATA1 expression and immune cell infiltration in DLBCL and showed that GATA1 expression was positively correlated with memory B cells, dormant CD4^+^ T cells, monocytes, and NK cells. In contrast, GATA1 expression was negatively correlated with dormant NK and CD8^+^ T cells (Figure 5(a)). TIMER, CIBERSORT, and CIBERSORT abs algorithms were used to verify the relationship between GATA1 expression and B cells, monocytes, NK cells, and neutrophils (Figure 5(b) and (c)). We then calculated the EstimateScore, ImmuneScore, and StromalScore of the two groups with high and low GATA1 expression (Figure 6(a) and (b)). The results showed that the immune core of the high-GATA1-expression group was significantly higher than that of the low-GATA1-expression group. Finally, we further explored the potential link between drug sensitivity and GATA1 expression using the CellMinerTM database (https://discover.nci.nih.gov/cellminer/home.do). Notably, GATA1 expression was positively correlated with sensitivity to tyrosine kinase inhibitors such as imatinib and nilotinib, and the ALK inhibitor crizotinib (Figure 7(a)), and significantly negatively correlated with PI3K inhibitors such as buparlisib, Src-Abl inhibitors, and the CDK9 inhibitor palbociclib (Figure 7(b)).

### 3.7 Verification of hub gene GATA1 expression by qPCR

**Figure 8:**
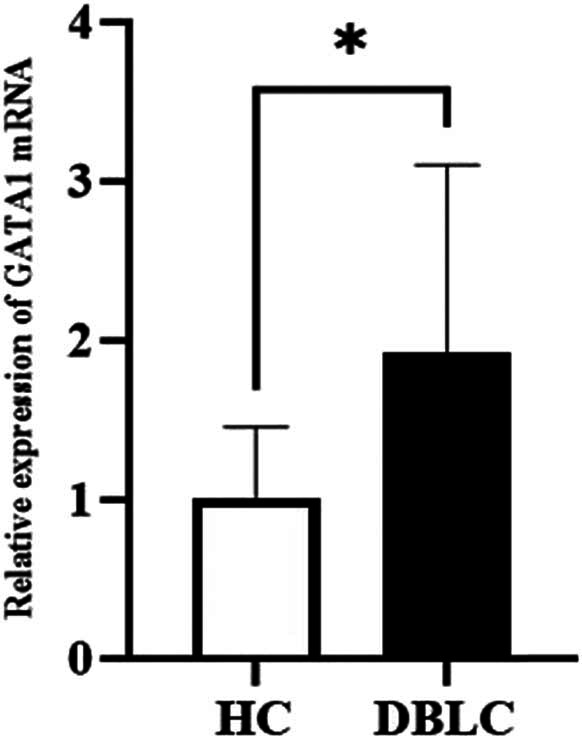
qPCR results about the expression levels of GATA1. A nonparametric Student’s *t*-test was calculated when comparing the two groups of HC and DLBCL. *P < 0.05.

Quantification of the mRNA abundance of GATA1 revealed that it was actively transcribed in the whole blood of DLBCL patients. Further expression levels of the GATA1 in PBMCs from HC and DLBCL patients were measured. Compared with HC, the relative expression levels of GATA1 mRNA were significantly increased in DLBCL patients (*P* < 0.05) (Figure 8).

## 4 Discussion

Increasing evidence suggests that ferroptosis is involved in the development of various tumors [19]; therefore, targeting iron-related cell death has substantial potential for tumor therapy [20]. To the best of our knowledge, this is the first study to apply a bioinformatics approach to explore the potential mechanisms underlying the association between iron metabolism and DLBCL, a highly aggressive and common subtype of non-Hodgkin lymphoma. First, we used the WGCNA algorithm, which is currently the most reliable algorithm for co-expression cluster analysis of iron metabolism and DLBCL (GSE83632) datasets. We then counted the intersections of genes in clinically relevant modules to calculate the shared genes. Simultaneously, we observed the biological processes and signaling pathways involved in these shared genes. Interestingly, the results of the enrichment analysis included multiple biological processes related to oxidative stress and apoptosis, which are closely related to DLBCL progression. These results suggest that the development of DLBCL may be related to transcriptional and apoptotic changes mediated by abnormal mitochondrial function [21]. To verify the authenticity of our data, the new DLBCL dataset GSE32918 (tissue RNA-seq results of DLBCL) and peripheral blood samples from the normal control group were screened again for limma analysis to detect differential genes, as well as their interaction with previously screened shared genes, to finally identify three core genes, GATA1, KLF1, and ACSL6 [22,23]. Considering the expression and prognosis of DLBCL, we identified GATA1 as a key gene involved in iron metabolism that affects DLBCL progression.

The GATA family consists of six transcription factors, GATA1–GATA6, known for their ability to bind to the DNA consensus sequence (A/T)GATA(A/G) through their characteristic zinc finger structure [24]. In our study, through the collation and analysis of DLBCL datasets from the TCGA and GTEx databases, we found that the group with high GATA1 expression had longer overall survival than the group with low GATA1 expression; high GATA1 expression also predicted a better prognosis. This finding was similar to that reported by Lin et al. who found that the expression level of GATA1 in acute promyelocytic leukemia was highest in the high-risk group and lowest in the low-risk group [25]. In 2022, Professor Zhang Huilai’s team published a paper in *Leukemia*, a hematology journal, confirming the role and mechanism of action of ferroptosis in the treatment of DLBCL and clarifying that ferroptosis is of great significance in the development and progression of DLBCL [26]. GATA1 belongs to the FerrDB. And it promotes cell invasion, metastasis, and drug resistance [27,28,29]. Therefore, the mechanism of action of GATA1 in DLBCL requires further exploration.

GATA1 plays an important role in regulating cell growth and development [30]. In this study, we analyzed the gene of GATA1 by three different immune infiltration databases and observed that GATA1 expression had a positive effect on CD4^+^ T cells, B cells, and monocytes, but a negative effect on NK cells and neutrophils. GO enrichment analysis showed that GATA1 was associated with myeloid differentiation and granulocyte differentiation pathways. It has been demonstrated that a high percentage of CD3^+^, CD4^+^, and CD8^+^ T cells in the tumor microenvironment of DLBCL correlates with a favorable prognosis [29]. By ssGSEA, we can see that there is an oxidative phosphorylation pathway. Oxidative phosphorylation closely affects the activation, proliferation, and cytokine secretion of CD8^+^ T cells in response to tumor antigen stimulation [31]. Modulation of the CD8^+^ T-cell response has been a central focus of immunotherapy for the treatment of tumors, and it is the immune cell of choice for targeting tumors [32]. Currently, chimeric antigen receptor T-cell immunotherapy (CART), an emerging hotspot therapy for DLBCL, *in vivo* CAR-T cell expansion is dominated by CD8^+^ T cells and is most significantly associated with durable responses in patients with relapsed/refractory DLBCL (r/r DLBCL) [33–35]. In 2024, Professor Yi Zhang’s team discovered that the PD-1 signaling pathway inhibits phospholipid phosphorylation (PLPP1) expression through the Akt–GATA1 pathway to inhibit PLPP1 expression, leading to impaired phospholipid metabolism, reduced anti-tumor capacity, and increased iron death in CD8^+^ T cells. It suggests that GATA1 is regulating the occurrence of ferroptosis in tumor-infiltrating CD8^+^ T cells, thus affecting the anti-tumor function to provide a new direction and theoretical basis for the study of ferroptosis in DLBCL with high GATA1 expression [36]. Therefore, we believe that GATA1 is of great significance in predicting the prognosis of patients with DLBCL.

Currently, the treatment of choice for DLBCL is chemotherapy; although most patients are sensitive to first-line chemotherapy, 30–40% of patients with DLBCL still relapse after treatment [37]. However, somebody cannot be treated with both chemotherapy or CART due to age and chemotherapy sensitivity, economic conditions, and physical status. Therefore, many investigators are continuously exploring other second-line salvage options. In our study, the peripheral blood of DLBCL patients was analyzed using PCR technique indeed GATA1 was highly expressed and statistically significant. Network pharmacological analysis reveals that GATA1 is strongly associated with sensitivity to small molecule targeted drugs. In particular, the expression of GATA1 was positively correlated with imatinib, nilotinib, and crizotinib sensitivity but negatively correlated with the PI3K inhibitor copanlisib, the Src-Abl inhibitor, and the CDK9 inhibitor [38]. The current study by Prof. Yi Zhang did find that the transcript GATA1 is indeed an upstream molecule of the AKT pathway. Thus, we speculate that GATA1 causes relapsed refractory DLBCL to become resistant to copanlisib by affecting the PI3K/Akt/mTOR pathway. However, the exact mechanism requires further investigation.

Our study has some limitations. That is, the results are still at the level of data analysis, and insufficient experimental data exist to confirm our results. Therefore, a reasonable analysis must be performed to verify our conjectures in a stepwise manner.

## 5 Conclusion

In conclusion, through WGCNA, modules related to iron metabolism and DLBCL were identified. After enrichment analysis of the modules, the key gene GATA1 was finally identified. The pathway of GATA1 in DLBCL was investigated and the relationship between GATA1 expression and immune cell infiltration in DLBCL was deduced. Moreover, the relationship between the expression of GATA1 and the sensitivity of DLBCL treatment was analyzed. This study provides novel insights into the molecular mechanism between iron metabolism and DLBCL. Specifically, we present GATA1 as a meaningful biological target and immune-related biomarker of DLBCL.
